# A Method for the Isolation and Culture of Adult Rat Retinal Pigment Epithelial (RPE) Cells to Study Retinal Diseases

**DOI:** 10.3389/fncel.2015.00449

**Published:** 2015-11-20

**Authors:** Janosch P. Heller, Jessica C. F. Kwok, Elena Vecino, Keith R. Martin, James W. Fawcett

**Affiliations:** ^1^John van Geest Centre for Brain Repair, Department of Clinical Neurosciences, University of CambridgeCambridge, UK; ^2^Department of Clinical and Experimental Epilepsy, Institute of Neurology, University College LondonLondon, UK; ^3^Department of Cellular Biology, University of the Basque CountryLeioa, UPV/EHU, Bizkaia, Spain; ^4^Department of Ophthalmology, NIHR Biomedical Research Centre and Wellcome Trust—Medical Research Council Cambridge Stem Cell Institute, University of CambridgeCambridge, UK

**Keywords:** retinal pigment epithelium, rat, papain, isolation, culture, adult, age-related macular degeneration, retina

## Abstract

Diseases such as age-related macular degeneration (AMD) affect the retinal pigment epithelium (RPE) and lead to the death of the epithelial cells and ultimately blindness. RPE transplantation is currently a major focus of eye research and clinical trials using human stem cell-derived RPE cells are ongoing. However, it remains to be established to which extent the source of RPE cells for transplantation affects their therapeutic efficacy and this needs to be explored in animal models. Autotransplantation of RPE cells has attractions as a therapy, but existing protocols to isolate adult RPE cells from rodents are technically difficult, time-consuming, have a low yield and are not optimized for long-term cell culturing. Here, we report a newly devised protocol which facilitates reliable and simple isolation and culture of RPE cells from adult rats. Incubation of a whole rat eyeball in 20 U/ml papain solution for 50 min yielded 4 × 10^4^ viable RPE cells. These cells were hexagonal and pigmented upon culture. Using immunostaining, we demonstrated that the cells expressed RPE cell-specific marker proteins including cytokeratin 18 and RPE65, similar to RPE cells *in vivo*. Additionally, the cells were able to produce and secrete Bruch’s membrane matrix components similar to *in vivo* situation. Similarly, the cultured RPE cells adhered to isolated Bruch’s membrane as has previously been reported. Therefore, the protocol described in this article provides an efficient method for the rapid and easy isolation of high quantities of adult rat RPE cells. This provides a reliable platform for studying the therapeutic targets, testing the effects of drugs in a preclinical setup and to perform *in vitro* and *in vivo* transplantation experiments to study retinal diseases.

## Introduction

The retinal pigment epithelium (RPE) is a highly polarized pigmented monolayer sandwiched between the photoreceptor cells and the choroid at the back of the eye (Strauss, 2005; Bertolotti et al., 2014). RPE cells serve essential roles in the healthy retina; they phagocytoze shed photoreceptor outer segments (POS) and recycle retinoids (Strauss, 2005; Bertolotti et al., 2014; Solinis et al., 2015). They are part of the blood-retina barrier and mediate the bidirectional transport between the neural retina and the blood vessels in the choroid (Strauss, 2005; Bertolotti et al., 2014). Moreover, RPE cells secrete factors important for the development and maintenance of the retina, the choriocapillaris and Bruch’s membrane (Strauss, 2005; Bertolotti et al., 2014).

Retinal diseases such as Leber congenital amaurosis and age-related macular degeneration (AMD) affect the RPE and lead to its malfunction and degeneration with associated visual loss (Heller and Martin, 2014; Pierce and Bennett, 2015; Sahel et al., 2015; Solinis et al., 2015). RPE cell transplantation has received much attention as a treatment for AMD. The transplantation of RPE cells prevented the progression of photoreceptor and visual loss in various animal models. However, attempts to transplant new RPE cells into diseased eyes of human AMD patients have been challenging (Algvere et al., 1994, 1997, 1999; Binder et al., 2002, 2004, 2007; Tezel et al., 2007; Falkner-Radler et al., 2011; Schwartz et al., 2012), and only resulted in improved vision in a limited number of cases (Heller and Martin, 2014).

Several types of RPE cells have been studied and used for transplantation experiments in animal models (Pfeffer and Philp, 2014), including cell lines (Coffey et al., 2002; Wang et al., 2005), fetal (Little et al., 1996, 1998) and adult human RPE cells (Castillo et al., 1997) as well as stem cell-derived RPE cells (Lund et al., 2006; Vugler et al., 2008; Carr et al., 2009; Lu et al., 2009). Besides human tissue-derived cells, RPE cells from various animal species have been used for the transplantation into animal models (Li and Turner, 1988). The use of homologous grafts has two main advantages: firstly, it avoids immunoreaction as a complication in animal transplantation studies. Secondly, animal tissue is more readily available in comparison to human tissue. Furthermore, the differentiation of human stem cells into functional RPE cells takes ~4–10 weeks without expansion (Idelson et al., 2009; Buchholz et al., 2013; Brandl et al., 2014; Lane et al., 2014). Therefore, the culture of primary animal cultures is less time and labor-intensive and provides sufficient numbers of cells to study retinal diseases *in vitro* as well as *in vivo*.

Although several protocols for the isolation of RPE cells from rat tissue have been reported (Table 1), most methods rely on the use of very young animals for the dissection (Edwards, 1977, 1981; Mayerson et al., 1985; Chang et al., 1991; Sakagami et al., 1995). Only four published approaches describe the isolation of RPE cells from adult rats (Sheedlo et al., 1993; Wang et al., 1993; Kreppel et al., 2002; Langenfeld et al., 2015). This is likely to be due to the fact that adult cells are very fragile and can easily get damaged during the isolation process. In particular, the separation of RPE cells from the overlying retina becomes increasingly difficult due to the interlinkage of RPE microvilli and rod outer segments (Wang et al., 1993). Three of the four publications, are based on the same principal; the incubation of sclera, choroid and RPE sheets in trypsin (Sheedlo et al., 1993; Kreppel et al., 2002; Langenfeld et al., 2015), and only Langenfeld et al. (2015) stated an achieved yield (13,000 cells/eye or 30,000 cells/eye). Moreover, Kreppel et al. (2002) used the cells only to test virus infection and the cells isolated using Wang et al.’s (1993) protocol were used for biochemical assays rather than culturing.

**Table 1:** **Comparison of published dissection methods of rat RPE cells with our current protocol**.

Age	Incubation	Dissection*	Yield**	Reference
P0	1. 15’ 37°C 20 U/ml papain	1. globe	N/S	Pinzon-Duarte et al. (2000)***
P6–8	1. 6–24 h RT BSS	1. globe	N/S	Edwards (1977)
	2. 45’ 37°C 0.1% trypsin	2. globe		
	3. 8–10’ 37°C 0.1% trypsin	3. RPE		
P6–8	1. 6-24 h RT BSS	1. globe	30,000 –	Edwards (1981)
	2. 45’ 37°C 0.1% trypsin + 70 U/ml collagenase	2. globe	60,000	
	3. 1–4’ RT 0.1% trypsin	3. RPE		
P6-8	1. 30’ 37°C 2% dispase	1. globe	65,000	Chang et al. (1991)
	2. 10–15’ 37°C DMEM	2. retina + RPE		
	3. 2–3’0.1% trypsin	3. RPE		
P6-15	1. 13–15’ 37°C 0.1% proteinase K	1. globe	40,000	Sakagami et al. (1995)
	2. 10’ 37°C medium	2. retina + RPE		
	3. 7’ 37°C 0.1% trypsin	3. RPE		
P8-18	1. 45–90’ 37°C 105 U/ml collagenase + 50 U/ml hyaluronidase	1. globe	30,000 –	Mayerson et al. (1985)
	2. 57–72’ 37°C 0.1% trypsin	2. globe	40,000	
	3. 1.5–2.5’ 37°C 0.1% trypsin	3. RPE		
Adult	1. 20’ 37°C 0.25% trypsin	1. sheet	N/S	Kreppel et al. (2002)****
4–14 weeks	1. 12’ 37°C 220 U/ml hyaluronidase type IV + 65 U/ml collagenase	1. sheet + retina	N/S	Wang et al. (1993)****
	2. 8’ 37°C 220 U/ml hyaluronidase type IV + 65 U/ml collagenase	2. sheet		
	3. 30’ RT CFHE	3. sheet		
8-10 weeks	1. 50’ 37°C 20 U/ml papain	1. globe	30,000 –	Our method
	2. 10’ 37°C 20 U/ml papain	2. retina + RPE	40,000	
	3. 20’ 37°C 1 mg/ml trypsin	3. RPE		
2-4 month, 17 month	1. 15–30’ 37°C 0.1% trypsin	1. sheet	N/S	Sheedlo et al. (1993)
6-14 weeks	1. 10’ 37°C 0.125% trypsin	1. sheet	13,000	Langenfeld et al. (2015)
	or	or	or	
	1. outgrowing cells after plating of sheet	1. sheet	30,000	

BSS, balanced salt solution; CFHE, calcium-free Hank’s EDTA; DMEM, Dulbecco’s modified Eagle’s medium; P, postnatal day; RT, room temperature; N/S, not stated. * = incubation of the whole eye in protease with subsequent dissection (globe) or dissection of the eye and incubation of sclera, choroid and RPE sheet in proteinase (sheet) with or without retina, or incubation of the RPE layer with or without retina. ** = yield per eye ball. *** = Pinzon-Duarte et al. (2000) did not culture RPE cells but isolated the retina with intact attached sheets of RPE cells. **** = Wang et al. (1993) and Kreppel et al. (2002) did not isolate RPE cells for extensive culturing but to test virus infection or to use the cells for biochemical assays, respectively.

To achieve a more reliable method and a higher yield from adult rats, we systematically developed and validated a new way to isolate RPE cells. Our protocol is fast, easy and efficient and would provide a valuable tool for the study of AMD and other diseases involving RPE pathology. Our culture method of rodent adult RPE cells is based on the digestion of the whole eye in papain. We demonstrated the success of the culturing protocol using two-month old rat eyes. However, the method is widely applicable to other animals, such as mice, with slight modifications. The protocol provides a useful RPE cell culture model which can be used to evaluate the behavior of adult RPE cells when subjected to certain stresses *in vitro*, giving a result more reminiscent to the disease situation. Also, the cultured cells can be transplanted directly in common animal models of retinal degeneration such as the Royal College of Surgeon’s (RCS) rat. We assessed our method by comparing cell yield, growth characteristics, the expression of RPE markers and the adhesion of the cells to Bruch’s membrane components to previous data reported in the literature.

## Materials and Methods

### RPE Cell Culture

All animal work was carried out in accordance with the UK Animals (Scientific Procedures) Act 139 (1986) and within UK Home Office regulations. Animal experiments and designs were covered under Project licence 80/2360 approved by the Home Office. Adult Lister Hooded or Sprague Dawley (SD) rats (Charles River; 250–300 g, 8–10 weeks) were used for tissue. The rats were kept in standard housing conditions with 12 h light/dark cycle in a temperature-controlled room (22°C) with free access to food and water.

**Figure 1:**
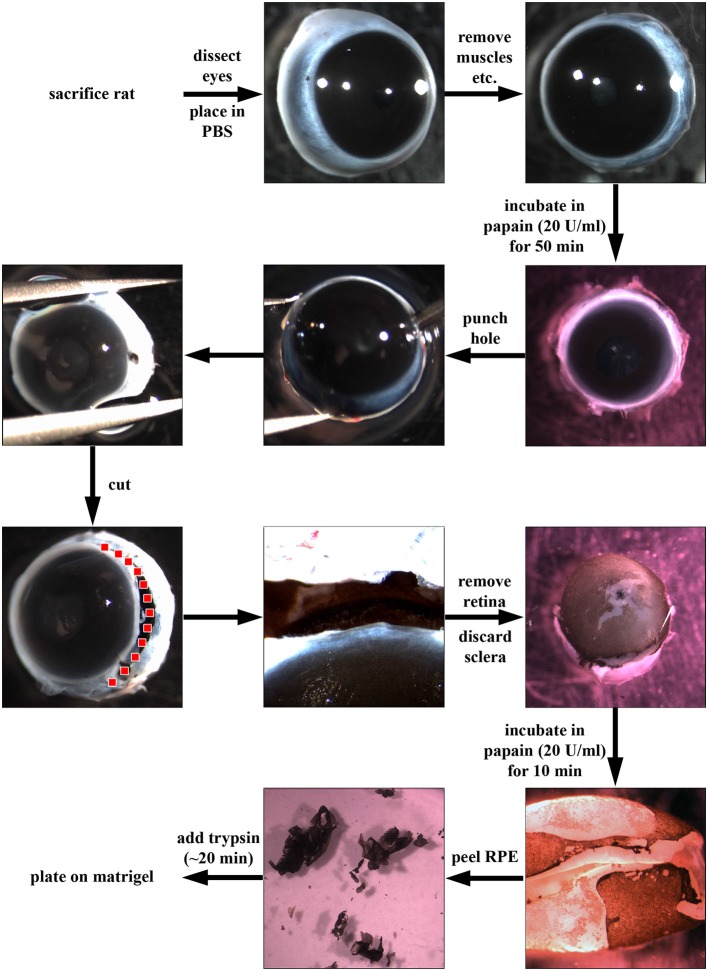
**Dissection of retinal pigment epithelial (RPE) cells using papain.** First, rats were sacrificed using CO_2_ and dislocation of the neck. Then, eyes were dissected out, placed in phosphate-buffered saline (PBS) and excess connective tissue and muscle attachments were removed. Afterwards, the whole eye was incubated in papain (20 U/ml, Worthington PDS Kit) for up to 1 h at 37°C. The papain digestion was stopped by the addition of DMEM supplemented with 10% fetal bovine serum (FBS). Using a needle, a hole was introduced into the globe near the ora serrata, and the anterior part of the eye was cut away (following the red dotted line). Afterwards, the retina was carefully removed, and the choroid-sclera sheet was discarded. The RPE cells were still attached to the retina (pigmented cells in figure). The retina was then incubated for another 10 min in papain (20 U/ml) to loosen the RPE sheets which were then peeled off and incubated in trypsin (1 mg/ml) and triturated to achieve a single cell solution. Finally, the RPE cells were plated in “Miller” medium (DMEM supplemented with 20% FBS, N1 medium supplement, MEM-non-essential amino acids, 2 mM GlutaMAX^™^-I, 250 μg/ml taurine, 20 ng/ml hydrocortisone, 13 ng/ml triiodothyronin and antibiotics) on matrigel (1:80 in DMEM)-coated dishes.

A detailed step-by-step guide to the process is illustrated in Figure 1 Rats were culled by exposure to rising concentration of CO_2_ followed by dislocation of the neck. Eyes were dissected out, and excess muscle and connective tissue were removed in ice cold phosphate-buffered saline (PBS). The eyes were then incubated in a 20 U/ml papain solution (Worthington PDS Kit) for up to 1 h at 37°C in a 24-well-plate. Four eyes can be placed in one well of the plate, and 1 ml of the papain solution should cover all eyes. Afterwards, eyes were transferred to DMEM supplemented with 10% fetal bovine serum (FBS) to stop the papain digestion. Using a needle (21 gauge, BD Microlance), a hole was introduced close to and an incision was made along the ora serrata to remove the lens and cornea-iris. The earlier digestion with papain allowed the retina to be pulled out readily, leaving the choroid-sclera complex behind. At this point, the RPE cells were still attached to the retina. The retina/RPE complex was further digested in 1 ml of fresh 20 U/ml papain for ~10 min at 37°C. By means of fine forceps, the RPE sheets were peeled off the retina. The sheets were incubated in trypsin (1 mg/ml in PBS, Sigma) and then triturated to achieve smaller patches of RPE cells. The trypsin cell solution was diluted with Dulbeccos modified Eagles medium (DMEM) supplemented with 10% FBS and washed through centrifugation. RPE cells (different cell numbers, see below) were plated on matrigel-coated (BD Biosciences; 1:80 in ice-cold DMEM and then incubated overnight at 37°C) culture dishes in “Miller” medium (DMEM supplemented with 20% FBS, N1 medium supplement, MEM-non-essential amino acids, 2 mM GlutaMAX^™^-I, 250 μg/ml taurine, 20 ng/ml hydrocortisone, 13 ng/ml triiodothyronin and antibiotics; Maminishkis et al., 2006; Sonoda et al., 2009). The next day, the medium was changed to “Miller” medium supplemented with only 5% FBS. Medium was changed twice a week.

### Immunochemistry

Animals were perfused transcardially with 4% paraformaldehyde (PFA, Sigma) in 0.1 M PBS, pH 7.2–7.4, after an overdose of general anesthetic [Euthatal (200 mg/ml solution, Rhône-Mérieux)]. Eyes were dissected out, and lens and anterior chamber were carefully removed together with the vitreous humor. Care has to be taken to not destroy or detach the retina. The eyes were immediately post-fixed in 4% PFA overnight at 4°C. The tissue was transferred into 30% sucrose (Sigma) in PBS, for cryoprotection. Sections of 14 μm thickness were cut on a cryostat (Cryostat Leica CM 3050S) and mounted on glass slides (Superfrost^®^ Plus, VWR). The sections were washed three times with PBS. Subsequently, the tissue was permeabilized and blocked using PBS supplemented with 0.3% Triton-X 100 (Vector labs; PBST) and 10% donkey serum (Sigma; PBST-S) for 1 h at room temperature (RT). Afterwards, sections were incubated in primary antibody solution (antibody diluted in 5% PBST-S (Table 2) overnight (~14–16 h) at 4°C. The next day, sections were washed three times for 5–10 min each with PBST followed by the addition of secondary antibody solution (Alexa488- or Alexa568-coupled donkey anti-rabbit or donkey anti-mouse (Invitrogen) diluted in PBST-S). After 1–2 h of incubation at RT, the tissue was incubated for 5 min in PBS supplemented with HOECHST-33342 (2 μg/ml, Sigma) to stain for nuclei. Afterwards, the sections were washed again three times for at least 10 min with PBST. Finally, the sections were sealed with a coverslip using Fluorosave^™^ (Calbiochem).

**Table 2:** **Primary antibodies used**.

Antigen	Host	Clone	Supplier	Product Code
Basigin	Rabbit	EPR4052	Abcam	ab108317
Collagen IV	Rabbit	pc*	Abcam	ab19808
CRALBP	Mouse	B2	Novus	NB100–74392
Cytokeratin 18	Mouse	C–04	Abcam	ab668
Fibronectin	Rabbit	pc	Sigma	F3648
Laminin	Rabbit	pc	Sigma	l9393
MERTK	Rabbit	pc	Abcam	ab95925
OTX-2	Rabbit	pc	Millipore	AB9566
RPE65	Mouse	401.8B11.3D9	Millipore	MAB5428
ZO–1	Rabbit	pc	Invitrogen	61–7300

All antibodies were IgG. *pc = polyclonal.

Cultured RPE cells [3, 7 or 14 days *in vitro* (DIV)] were fixed with 4% PFA for 15 min at RT and washed three times with PBS. Subsequently, the cells were stained using the same protocol as for tissue sections.

To visualize the secreted extracellular matrix (ECM) molecules, RPE cells were cultured on poly-D-lysine (PDL)-coated glass coverslips overnight. The next day, cells were lysed with deionized water by osmosis and cell debris was squirted away. The coverslips were washed in PBS and stained for ECM molecules including collagen IV, fibronectin and laminin (Table 2) overnight at 4°C. Then, the primary antibodies were visualized using secondary antibodies (Alexa488-coupled donkey anti-rabbit, see above), and the coverslips were mounted onto slides using Fluorosave^™^ (Calbiochem), dried in the dark overnight, stored at 4°C or viewed under the microscope directly.

### Quantification of RPE Marker Expression In Cultured RPE Cells

RPE cells were cultured for 3, 7 and 14 DIV. At each timepoint, RPE markers were visualized by immunofluorescence. Images were acquired by fluorescence microscopy. Identical conditions for immunostainings were used within each experiment and images were acquired with identical microscope settings. Experiments were repeated three times and each time, at least 30 cells per group were measured in each experiment.

Images were processed using ImageJ. Cells were traced with the freehand selection tool, and mean fluorescence intensity was measured. After background subtraction, fluorescent intensity was averaged across cells. Statistical analysis was performed using one-way *ANOVA* with Dunnett’s *post hoc* test using GraphPad Prism software. The results are presented as mean + SEM (standard error of the mean). Significance values were represented as: *P < 0.05, **P < 0.01 and ***P < 0.001.

### RPE Adhesion to ECM Molecules Present in the Bruch’s Membrane

Glass coverslips (13 mm, acid-washed) were coated with collagen I, collagen IV, fibronectin or laminin (1 μg/ml, Sigma) for at least 2 h at RT. The coverslips were then washed twice with sterile PBS. Cultured RPE cells were briefly trypsinized (~3 min at 37°C), pelleted, washed and resuspended in Miller medium to a final concentration of 100,000 cells/ml. 500 μl (28,000 cells/cm^2^) of this solution were added to each coverslip in a well of a 24-well-plate. The plates were then incubated in a shaking incubator (Luckham R300) at 10 rounds per minute at 37°C for 1 h. After the incubation, the coverslips were washed three times with PBS to wash away loose cells. The attached cells were then visualized and counted under phase contrast microscopy (Nikon). Five random fields (at left, right, middle, top and bottom of coverslip) were chosen from each coverslip and the number of attached cells was counted. The average number of cells adhering was counted and normalized to the average number of attached cells under control conditions (non-coated glass coverslip). Each condition contained three coverslips and experiments were repeated three times. All data was analyzed using one-way *ANOVA* with Dunnett’s *post hoc* test using GraphPad Prism software. The results are presented as mean + SEM. Significance values were represented as: **P < 0.01.

## Results

### Development of the Adult RPE Culture Protocols

Most published protocols facilitate the isolation of RPE cells from very young rats (Table 1, Edwards, 1977, 1981; Mayerson et al., 1985; Chang et al., 1991; Sakagami et al., 1995). Only four publications describe the dissection of RPE cells from adult animals (Sheedlo et al., 1993; Wang et al., 1993; Kreppel et al., 2002; Langenfeld et al., 2015). The protocol we describe here (Figure 1) yielded the best results when compared directly to other published methods. Our protocol is based on a combination of methods, including the isolation of rat and mouse retina explants for electrophysiological measurements (Pinzon-Duarte et al., 2000; Agulhon et al., 2007) and the dispase-based protocol for the culture of RPE cells isolated from young animals (rats, mice and rabbits; Chang et al., 1991; Gibbs and Williams, 2003; Cong et al., 2008).

**Figure 2:**
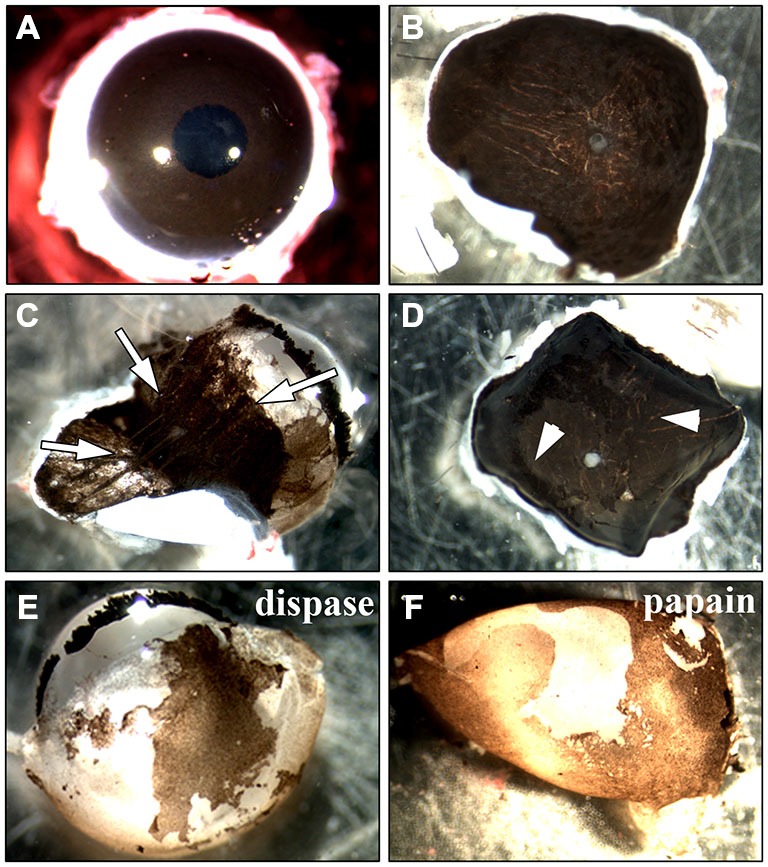
**Methods used for the isolation of RPE cells.** Two main approaches to isolate RPE cells from adult rat eyes have been tested. **(A)** The first approach involved the incubation of the whole eye in proteinase (Edwards, 1977, 1981; Mayerson et al., 1985; Chang et al., 1991; Sakagami et al., 1995; Pinzon-Duarte et al., 2000). After the incubation, the eye was opened and the anterior parts including the retina were removed. **(B)** For the second method sclera/choroid/RPE sheets were incubated in protease (Wang et al., 1993; Kreppel et al., 2002). **(C)** Trypsin, collagenase or hyaluronidase all dissolved the eye tissue so that the choroid became disintegrated and an isolation of RPE cells was impossible (white arrows). The same happened when the sclera/choroid/RPE sheet was incubated in these proteinases as has been commonly used for human tissue (Maminishkis et al., 2006; Gullapalli et al., 2008; Sonoda et al., 2009; Pfeffer and Philp, 2014). **(D)** Reducing the incubation time in trypsin, collagenase or hyaluronidase kept the choroid intact but in most cases, the pigmented RPE cells were still attached to Bruch’s membrane so that it was impossible to peel off the cells (see arrowheads). The incubation of the sheet in dispase, accutase or papain did not lead to an appropriate digestion of Bruch’s membrane and many RPE cells stayed attached to the underlying tissue. **(E)** Incubating the whole eye in 2% dispase (Chang et al., 1991) yielded ~25,000 cells/eye after 50 min. **(F)** Papain (20 U/ml) digestion resulted in the isolation of ~40,000 cells after 50 min (see Figure 1).

To find the best culture method for adult RPE cells, we first tried an incubation of the whole globe of an adult rat (Figure 2A) in (1) trypsin, (2) collagenase or (3) trypsin + collagenase as has been published for eyes dissected from 6–8 day old rats (Edwards, 1977, 1981). We also tried incubating the whole eye in collagenase followed by hyaluronidase (Mayerson et al., 1985). After the incubation, the eye was cut open and the anterior parts including the retina were removed to expose and remove the RPE cells. However, the pigmented RPE cells were still attached to the underlying choroid so that it was impossible to peel off the cells (white arrows in Figure 2D are pointing towards pigmented RPE cells). Changing the incubation time did not yield better results as a longer incubation resulted in dissolving the tissue boundaries so that the RPE cells were washed away with the removal of the retina (arrows in Figure 2C).

Besides incubating the whole globe we also tried the isolation of a sclera/choroid/RPE cell sheet with subsequent incubation in protease (Figure 2B). It was impossible to either peel off the RPE cells from the choroid directly, as described for human tissue (Hu and Bok, 2010), or to peel the choroid of the underlying sclera without destroying the tissue. Therefore, we did not incubate isolated choroid/RPE sheets but rather flattened posterior eye cups. First, we used either dispase (Castillo et al., 1995; Maminishkis et al., 2006; Blenkinsop et al., 2013) or papain (Kasahara et al., 2005; Bian et al., 2007). Nevertheless, the use of these proteases on the exposed RPE did not lead to a digestion of the Bruch’s membrane and did not allow the isolation of RPE cells because the cells were still tightly bound to the underlying choroid (Figure 2D). Using collagenase (Gullapalli et al., 2008), collagenase + hyaluronidase (Wang et al., 1993) or trypsin (Baumgartner et al., 1989; Hunt et al., 1989; Sheedlo et al., 1993; Kreppel et al., 2002; Klettner and Roider, 2008; Langenfeld et al., 2015) deteriorated tissue boundaries and the RPE cell layer could not be separated (Figure 2C). Also the use of forceps or small brushes to brush of the cells did not result in the separation of the RPE cells from the underlying choroid.

Only incubation of the whole eye in 2% dispase (Chang et al., 1991) or 20 U/ml papain (see below) led to isolation of RPE cells (Figures 2E,F). However, the incubation of the whole eye in dispase yielded fewer cells when compared to papain after 50 min (25,000 vs. 40,000 cells/eye).

We did not try the isolation using proteinase K described by Sakagami et al. (1995) as this protease is three times more active than trypsin (Ebeling et al., 1974) and trypsin resulted in dissolution of tissue (see above).

### Optimal Digestion Time for RPE Using Papain

The whole eye was digested in papain (20 U/ml) for 50 min before the retina was carefully removed (Figure 1). The RPE cells stayed attached to the retina, and the pigmented cells were peeled off as a discrete and coherent layer from the back of the retina following a second incubation in papain (20 U/ml) for 10 min (Figure 1). The RPE cell layer was then broken into smaller pieces through incubation in trypsin with subsequent trituration and cultured on matrigel in Miller medium. 30,000–40,000 cells were isolated from one eye using this method (Table 1).

**Figure 3:**
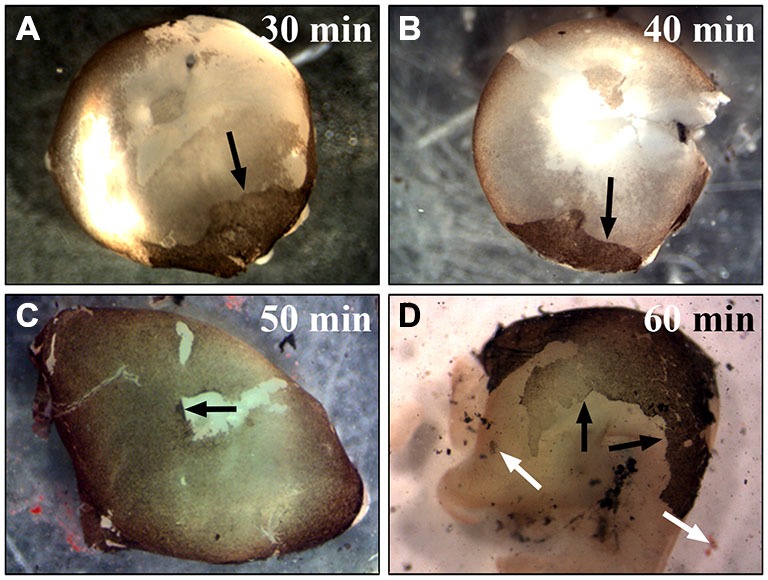
**Incubation of the whole eye in papain for different times.** To establish the best time point for the most efficient RPE isolation using papain the whole eye was incubated in papain solution (20 U/ml) for 30–60 min and longer. **(A)** After half an hour in papain, most RPE cells were still attached to the Bruch’s membrane, and with the removal of the retina only a small portion of the cells was removed from the eye (see arrow). **(B)** Digesting the eye for 40 min also only isolated a small number of RPE cells (see arrow). **(C)** The digestion of the whole eye for 50 min yielded the highest number of RPE cells (see arrow). **(D)** After 1 h or more the tissue started to get dissolved, and this resulted in a lower yield of RPE cells. The black arrows are pointing towards the attached RPE cell layer. The white arrows highlight the dissociated RPE tissue that was lost during the isolation.

To establish a time point that provided most efficient papain digestion, the eyes were kept in the papain solution for 30, 35, 40, 45, 50, 55, 60 min (Figure 3). We also tested different papain concentrations for the digestion of the whole eye. Using less than 20 U/ml of papain did not digest the Bruch’s membrane and the RPE cells remained tightly attached to the membrane even with digestions for more than 50 min (similar to Figure 3D). The use of more than 25 U/ml resulted in a quicker digestion of the eye. However, the RPE sheets were dissolved in this process and hardly any RPE cells were harvested. This high concentration also led to dissolution of the retina and the choroid (similar to Figures 2C, 3D). After 30–45 min incubation in 20 U/ml papain, most of the RPE cells were still stuck to Bruch’s membrane and only a small part of the RPE monolayer was removed from the eye together with the removal of the retina (see arrows in Figures 3A,B). Digestion of the whole eye for 45–55 min yielded the highest number of RPE cells stuck to the retina (see arrows in Figure 3C). Keeping the eye in papain for 1 h or even longer resulted in the dissolution of the tissue with a low yield of RPE cells (Figure 3D; black arrows are pointing towards RPE cell layer and white arrows at dissolving tissue). The above method is widely applicable for the isolation of RPE cells from eyes of different sizes (e.g., mice eyes). However, the incubation time has to be adjusted accordingly.

### The Expression of RPE Cell Markers in the Cultured RPE Cells

**Figure 4:**
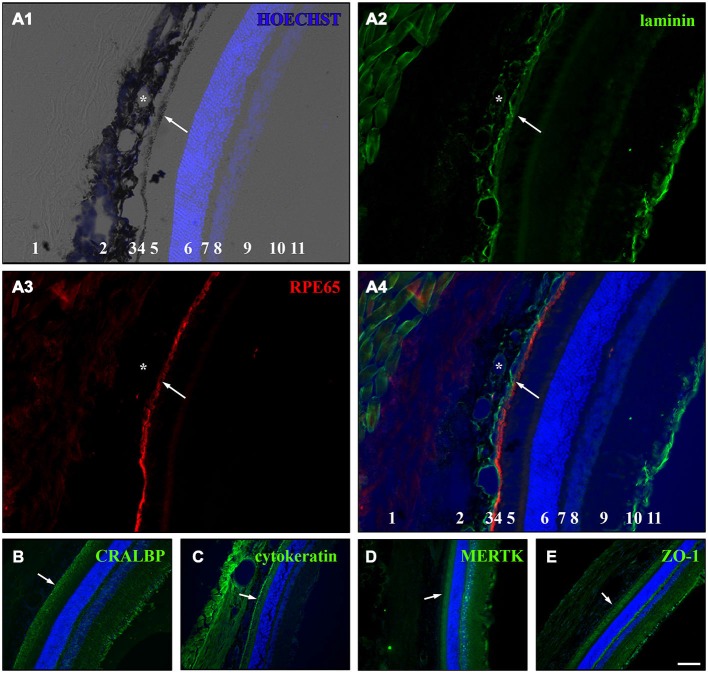
**Immunostaining for RPE cell markers in whole eye cross sections.** Ten week old LH rats were perfused with 4% PFA before the eyes were cryosectioned (14 μm). Afterwards, immunostaining was used to visualize several RPE cell markers in the eye sections (Table 2). HOECHST was used to illuminate cell nuclei (blue). **(A)** Staining for laminin (green) and RPE65 (red) in cryosections of LH rat. Numbers in A1 and A4: 1 = sclera, 2 = choroid, 3 = Bruch’s membrane, 4 = RPE, 5 = photoreceptor outer segments (POS), 6 = outer nuclear layer (ONL), 7 = outer plexiform layer (OPL), 8 = inner nuclear layer (INL), 9 = inner plexiform layer (IPL), 10 = retinal ganglion cell (RGC) layer, 11 = inner limiting membrane (ILM). **(A1)** Merge picture of brightfield and HOECHST (blue). The pigmented choroid (2) and RPE cell layer (4) are clearly evident in the brightfield image. The lumina of the blood vessels (asterisk) are visible in the choroid (2). The HOECHST staining visualizes the ONL (6) and the INL (8) with some faint cells visible in the RGC layer (10). **(A2)** Immunostaining for laminin (green) highlights the blood vessels (asterisk) within the choroid (2), the Bruch’s membrane (3) and the ILM (11). Also connective tissue outside the eye is stained for laminin. **(A3)** The monolayer of RPE cells (4) is strongly visible in addition to faint background staining in the sclera (1) and the region of the POS (4) when the cryosections were stained for RPE65 (red). **(A4)** In the merge image of the HOECHST (blue), laminin (green) and RPE65 (red) staining, it becomes clear that the Bruch’s membrane is immunopositive for laminin and is located adjacent to the RPE cell layer which is sandwiched between the choroid and the POS. **(B)** RPE as well as Müller cells expressed cellular retinaldehyde binding protein (CRALBP, green) which was especially visible in the Müller end feet that are part of the ILM. **(C)** Immunostaining for cytokeratin 18 (green) illuminated the RPE cells and also led to staining in the sclera and choroid. **(D)** The c-mer proto-oncogene tyrosine kinase (MERTK, green) was expressed by RPE cells and cells in or adjacent to the INL and the ILM. **(E)** The tight junction marker zona occludens protein 1 (ZO-1, green) was expressed in the RPE cells and in cells in the ILM. The white arrows are pointing towards the RPE cell layer in (**A–E**). Scale bar = 100 μm.

**Figure 5:**
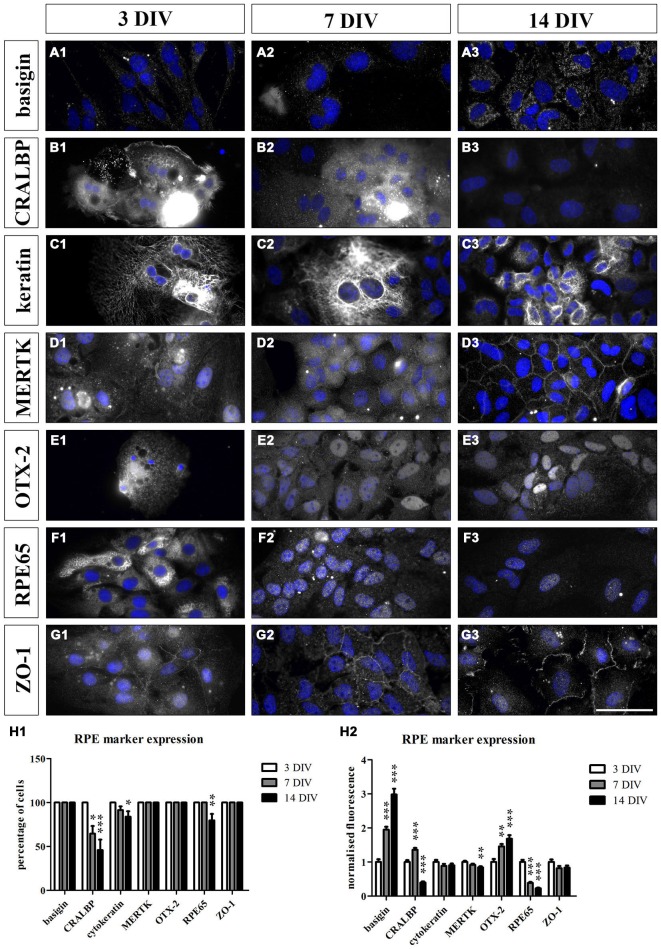
**Cultured RPE cells expressed RPE-specific markers.** We sparsely cultured 3000 fresh sprague dawley (SD) RPE cells per cm^2^ isolated using papain for 3, 7 and 14 days. We used immunostaining to evaluate whether the cultured cells lose their RPE-specific markers over time. At each timepoint, the cells were fixed with 4% PFA and stained for the respective cell markers (Table 2). Additionally, HOECHST was used to visualize cell nuclei (blue). We counted the number of cells that expressed the markers and compared the fluorescence intensity at all timepoints. **(A1–G1** and **H1)** The RPE cells expressed all the markers at 3 DIV. **(A1–A3** and **H1–H2)** Basigin was visible on the surface of the cells at all three timepoints and its expression increased during the culture period **(H2)**. **(B1–B3** and **H1–H2)** CRALBP was very prominent at 3 DIV **(B1)**. However, fewer cells expressed the marker over time **(H1)** and its expression level decreased and was almost completely lost at 14 DIV **(B3** and **H2)**. **(C1–C3** and **H1–H2)** Cytokeratin 18 was present in the RPE cells throughout the whole experiment. However, a small portion of the cells did not express this cytoskeletal marker at 7 and 14 DIV **(H1)**. **(D1–D3** and **H1–H2)** MERTK was expressed by the RPE cells at all three timepoints. The protein was especially prominent at the cell-cell contacts within the tight RPE cell monolayer that was formed at the end of the experiment **(D3)**. However, the expression intensity decreased over time **(H2)**. **(E1–E3** and **H1–H2)** The transcription factor OTX-2 was visible within the cytoplasm as well as the nucleus of the RPE cells at all three timepoints. Moreover, its expression level increased during the culture period **(H2)**. **(F1–F3** and **H1–H2)** RPE65 was very prominent in the RPE cells at 3 DIV (F1) but its expression decreased during the experiment **(H1** and **H2)**. **(G1–G3** and **H1–H2)** ZO-1 was visible at the margins of the hexagonally shaped RPE cells at all three timepoints. Scale bar = 50 μm.

To test whether the cultured RPE cells express RPE-specific markers, we cultured fresh RPE cells in low density (4000 cells per coverslip) for 3, 7 and 14 days on laminin-coated (10 μg/ml) coverslips (13 mm = ~0.41 cm^2^). The cells were fixed with 4% PFA at the end of each timepoint and stained for RPE cell markers (Table 2). We used basigin (CD147, EMMPRIN; Philp et al., 2003), cellular retinaldehyde binding protein (CRALBP; Huang et al., 2009), cytokeratin 18 (Johansson et al., 2010), c-mer proto-oncogene tyrosine kinase (MERTK; Feng et al., 2002; Nandrot et al., 2012), OTX-2 (orthodenticle homeobox 2; Martinez-Morales et al., 2003; Housset et al., 2013), RPE65 (65 kDa RPE specific protein; Huang et al., 2009; Johansson et al., 2010) and tight junction protein 1 (zona occludens protein 1, ZO-1; Konari et al., 1995; Campbell and Humphries, 2012) as markers for RPE cells in culture (Figures 4, 5). Immunochemistry in 14 μm thick eye sections of two-month old Lister Hooded rats was used as a control to evaluate the specificity of the antibodies (Figure 4). In these animals, the RPE cells are visible as a pigmented monolayer sandwiched between the pigmented choroid (the white asterisk highlights the lumen of a choroidal blood vessel in Figure 4A) and the POS (Figure 4A; the white arrow is pointing towards the RPE cells). Furthermore, staining for laminin reveals its presence in Bruch’s membrane as well as the choroid, the sclera and the inner limiting membrane (ILM; Figure 4A). The RPE cells express RPE65 (Figure 4A), CRALBP (Figure 4B), cytokeratin 18 (Figure 4C), MERTK (Figure 4D) as well as ZO-1 (Figure 4E) *in vivo*.

For the immunostaining of cultured RPE cells, we used SD rat-derived cells to avoid autofluorescence from the pigments as these rats are albino. The cultured RPE cells expressed all the markers at 3 DIV (Figures 5A1–G1,H1,H2). Basigin was visible on the surface of the cells at all three timepoints (Figures 5A1–A3,H1,H2). During the culture period, the expression of the protein increased (Figure 5H2). On the other hand, the expression of CRALBP was very prominent at 3 DIV (Figure 5B1) but decreased progressively during the culture period and was almost absent at 14 DIV (Figures 5B3,H2). Apart from the expression level, there were also fewer cells expressing this RPE marker the longer the cells were in culture (Figure 5H1). The RPE cells expressed the cytoskeletal protein cytokeratin 18 throughout the whole experiment (Figures 5C1–C3,H1,H2). However, some cells did not express cytokeratin when sparsely cultured (Figure 5H1). MERTK was visible in the cultured cells at all three timepoints (Figures 5D1–D3,H1,H2) but its expression level decreased at 14 DIV (Figure 5H2). The protein was especially prominent at the surface of the cells within the tight RPE cell monolayer that was formed at the end of the experiment (Figure 5D3). The transcription factor OTX-2 was visible in the cytoplasm as well as the nucleus of the RPE cells and its expression increased upon culturing (Figures 5E1–E3,H1,H2). The expression of RPE65 decreased during the experiment (Figures 5F1–F3,H1,H2). At 3 DIV, RPE65 was clearly visible in the cytoplasm of the cells (Figure 5F1). However, the protein was hardly detectable at 7 DIV and 14 DIV (Figures 5F2,F3,H2). Also the tight junction marker ZO-1 was expressed by RPE cells (Figures 5G1–G3,H1,H2). The protein was highly visible at the margins of the hexagonally shaped RPE cells at all three timepoints (Figures 5G1–G3). These results suggested that the cultured cells resembled endogenous RPE cells with similar RPE marker expression profiles.

### The Effect of Cell Density

**Figure 6:**
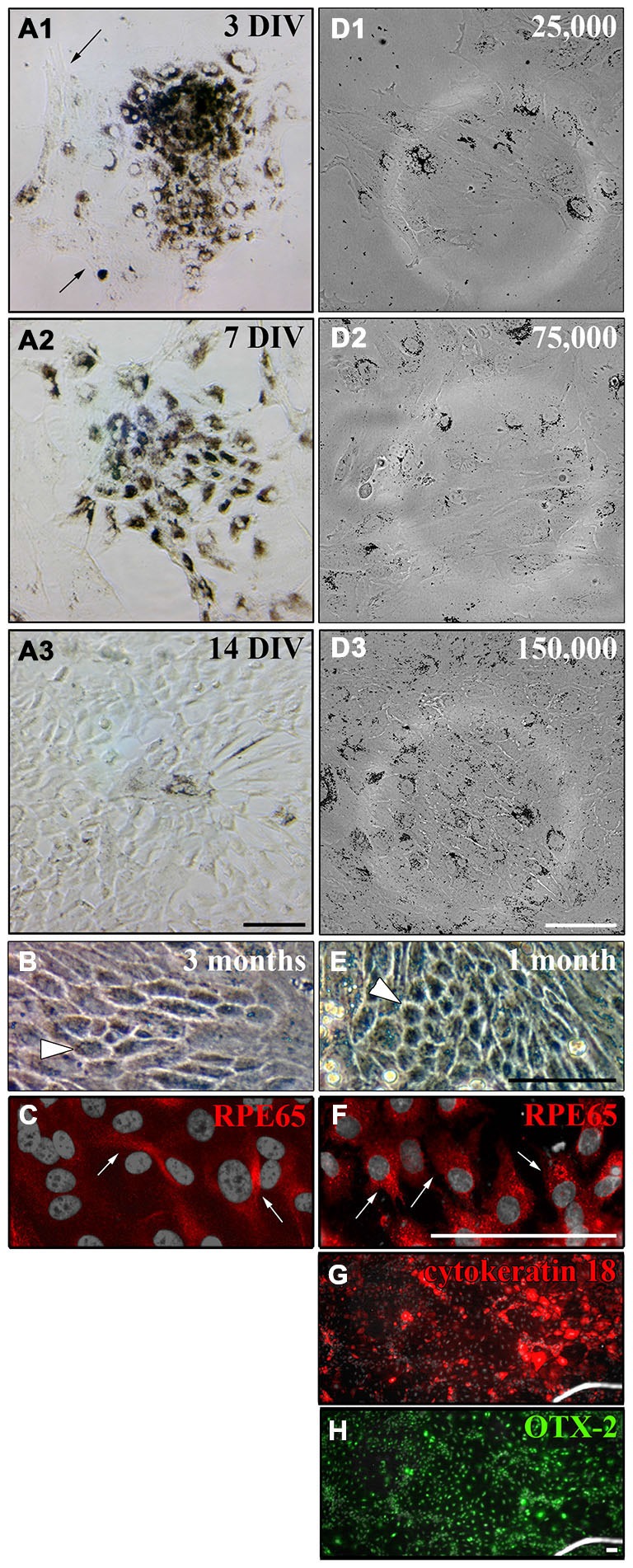
**Culture of primary adult RPE cells. (A)** Primary RPE cell patches (50–100 cells/patch and ~3500 cells/cm^2^) were dissected from adult LH rat eyes using incubation of the whole eye in papain for 50 min (20 U/ml) and plated in “Miller” medium on matrigel (1:80 in DMEM). **(A1)** Three days after dissection, the RPE cells started to proliferate and unpigmented cells appeared around the RPE cell patch (see arrows). **(A2)** After 7 days *in vitro* (DIV) the initial RPE cell patch appeared dissolved and the cells covered most of the culture dish. **(A3)** Although the cells lost their pigments they formed a tight monolayer throughout the culture dish after 2 weeks. **(B)** Only a few cells either stayed or became pigmented again even after 3 months in culture when ~3500 cells/cm^2^ were plated (white arrowhead). **(C)** Only a few pigmented cells in culture expressed RPE65 when ~3500 cells/cm^2^ were plated (white arrows, see Figure 5). **(D)** RPE cells were prepared using papain, and 25,000 (~2667 cells/cm^2^), 75,000 (~8000 cells/cm^2^) or 150,000 cells (~16,000 cells/cm^2^) were plated in a well of a 6-well-plate. **(D1** and **D2)** Plating ~2667 or ~8000 cells/cm^2^ led to a more spread-out morphology of the cells. The tightly packed architecture of the RPE monolayer got destroyed. **(D3)** The RPE cells stayed pigmented and tightly packed as a monolayer when ~16,000 cells/cm^2^ were plated. **(E)** Plating more cells initially (~16,000 cells/cm^2^) resulted in a tighter and more pigmented monolayer after 1 month in culture (white arrowhead). **(F)** Almost all cells expressed RPE65 when more cells were plated initially (white arrows). **(G** and **H)** All cells in culture expressed cytokeratin 18 and OTX-2. Scale bar = 100 μm.

As the cultured RPE cells lost the expression of CRALBP and RPE65 over time (Figures 5B3,F3), we wanted to test whether the cells also lose their pigmentation and whether this could be influenced by the cell density. RPE cell patches (50–100 cells/patch and ~3500 cells/cm^2^) attached to matrigel-coated (1:80 in DMEM) flasks and started to proliferate shortly after plating. After 3 DIV the cells started to proliferate and migrate away from the cell patch (Figure 6A1). One week after dissection, the RPE cell patch appeared dissolved, the cells migrated and proliferated forming an almost complete monolayer. However, most of the cells in the culture lost their pigmentation (Figure 6A2). The cells formed a non-pigmented monolayer after 2 weeks *in vitro* (Figure 6A3). Even after 3 months in culture, only a few cells were pigmented (white arrowhead in Figure 6B) and expressed RPE65 (Figure 6C).

It has been shown that plating RPE cells in low density leads to dedifferentiation of the cells when cell-cell contacts are lost (Sheridan et al., 2004; Kim et al., 2008; Tamiya et al., 2010). Therefore, we investigated the effect of initial cell density on the pigmentation and de-differentiation of RPE cells after culture (Figures 6D–F). When 25,000 or 75,000 cells were plated into a well of a 6-well-plate (Nunc; 9.6 cm^2^/well; ~2667 or ~8000 cells/cm^2^), the cells adapted a more spread-out morphology and the tightly packed architecture of the RPE monolayer got destroyed 3 days after plating (Figures 6D1,D2). However, the RPE cells stayed pigmented and tightly packed as a monolayer when 150,000 cells (~16,000 cells/cm^2^) were used (Figure 6D3). Hence, plating ~16,000 cells/cm^2^ allowed the RPE cells to proliferate and to form a monolayer after 2 weeks that stayed pigmented with hexagonal cells visible for at least 1 month in culture as seen *in vivo* (Burke and Hjelmeland, 2005; Figure 6E). Most of the cells expressed RPE65 (Figure 6F) and all of the cells expressed cytokeratin 18 and OTX-2 (Figures 6G,H) when ~16,000 cells/cm^2^ were plated.

### Secretion of Bruch’s Membrane Components *in Vitro*

**Figure 7:**
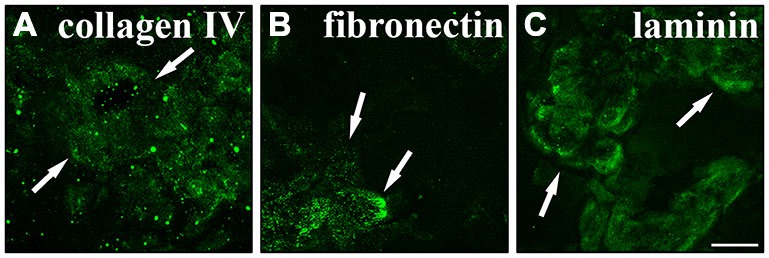
**Secretion of normal Bruch’s membrane components by RPE cells.** RPE cells (15,000 cells/cm^2^) cultured for 2 weeks were seeded on poly-D-lysine (PDL)-coated coverslips overnight and lysed the next day using deionized H_2_O. Using immunostaining the underlying matrix was visualized. RPE cells expressed and secreted collagen IV **(A)**, fibronectin **(B)** and laminin **(C)**. White arrows are pointing at sites of strong immunoreactivity. Scale bar = 50 μm.

One important function of RPE cells is the secretion and replenishment of ECM components for the integrity of Bruch’s membrane (Booij et al., 2010; Sato et al., 2013). In particular, the ECM molecules collagen, fibronectin and laminin are generated by RPE cells and make up the main components of the basement membrane and the inner collagenous layer of Bruch’s membrane (Booij et al., 2010; Sato et al., 2013). Therefore, we investigated whether RPE cells were capable of secreting their own ECM in culture. RPE cells (15,000 cells/cm^2^) were grown in culture for 2 weeks on matrigel. Then, the cells were trypsinized and re-plated on PDL-coated coverslips overnight and lysed on the next day using deionized water. This will reveal the ECM molecules secreted and deposited on the coverslips by the cultured cells (Afshari et al., 2010a,b). Immunostaining to visualize collagen IV (Figure 7A), fibronectin (Figure 7B) and laminin (Figure 7C) revealed that the cultured adult RPE cells expressed and deposited these fundamental Bruch’s membrane components in our experimental model. These findings are in agreement with previous studies investigating the secretion of ECM molecules by RPE cells, especially to form Bruch’s membrane *in vivo* (Campochiaro et al., 1986; Aisenbrey et al., 2006; Afshari et al., 2010a; Booij et al., 2010; Sato et al., 2013).

### Adhesion to Bruch’s Membrane Components

Next, we evaluated whether the cultured RPE cells were able to bind to Bruch’s membrane components. This would provide the important information if the cells are suitable for investigating the behavior of transplanted RPE cells in an *in vitro* situation. To this end, we performed cell adhesion assays on glass coverslips coated with 1 μg/ml of the Bruch’s membrane ECM molecules collagen I, collagen IV, fibronectin and laminin. Non-coated glass coverslips served as controls. RPE cells were trypsinized and seeded onto the coverslips in 24-well-plates (28,000 cells/cm^2^; Afshari et al., 2010a). Immediately after plating, the plates were incubated in a shaking incubator (10 rounds per minute) at 37°C for 1 h. After the incubation, the coverslips were washed three times with PBS to remove the unbound, loose cells. The attached cells were then counted in five random fields under phase contrast microscopy.

**Figure 8:**
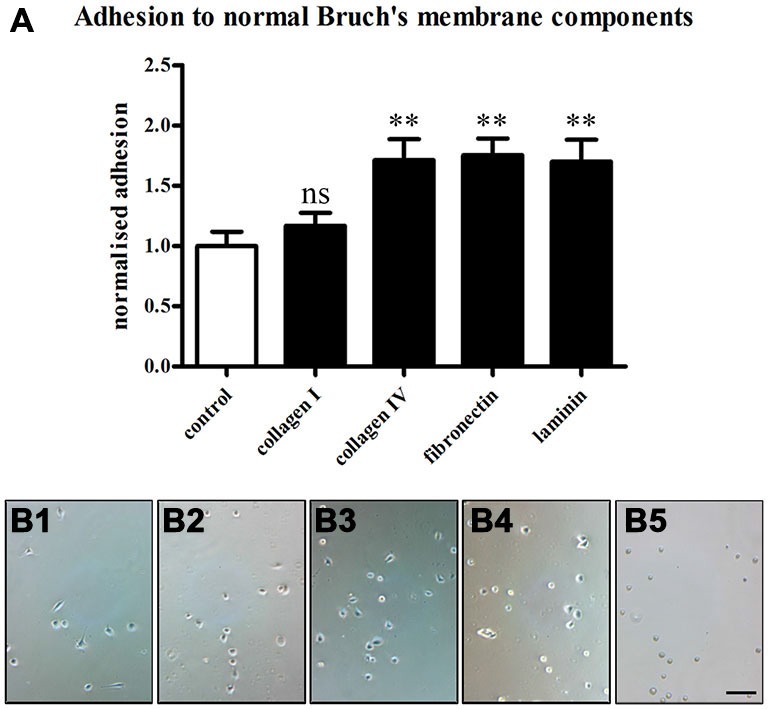
**Adhesion of RPE cells to normal Bruch’s membrane components.** RPE cells (28,000 cells/cm^2^) were seeded on the normal Bruch’s membrane components collagen I, collagen IV, fibronectin and laminin (1 μg/ml), and 1 h adhesion assays were carried out. Afterwards, the coverslips were washed three times with PBS to remove unbound cells, and attached cells were counted under phase contrast. **(A)** The Bruch’s membrane components promoted the adhesion of the RPE cells in the order of fibronectin > collagen IV > laminin > collagen I > non-coated. The number of RPE cells binding to collagen IV, fibronectin and laminin was significantly increased compared to control (*ANOVA*: ** = *P* < 0.01, ns = non-significant). **(B)** Adhesion assay on control non-coated glass coverslips **(B1)** and in collagen I- **(B2)**, collagen IV- **(B3)**, fibronectin- **(B4)** or laminin-coated **(B5)** conditions. *n* = 3. Scale bar = 200 μm.

The RPE cells showed different behaviors depending on the ECM molecules they were seeded on (Figure 8). The ECM molecules promoted the attachment of the RPE cells in the order of fibronectin (26.53 ± 2.1) > collagen IV (25.91 ± 2.62) > laminin (25.73 ± 2.77) > collagen I (17.69 ± 1.62) > non-coated (15.13 ± 1.81; mean ± SEM; *ANOVA*: ** = *P* < 0.01, ns = non-significant; Figure 8A). These findings are in agreement with earlier studies that showed the differential binding of human RPE cells to the different layers of Bruch’s membrane (Ho and Del Priore, 1997; Wang et al., 2003, 2006; Gullapalli et al., 2004; Afshari et al., 2010a).

## Discussion

Here, we describe a detailed protocol for an easy isolation and culture of adult rat RPE cells. These cells express RPE-specific marker proteins and form a hexagonal monolayer in culture. Moreover, the cells secrete the Bruch’s membrane components collagen IV, fibronectin and laminin. Additionally, the cultured RPE cells are able to adhere to Bruch’s membrane components in culture.

To find the best method for the culture of RPE cells, we systematically tried several published protocols (Table 1). Most methods described the dissection of RPE cells from neonatal or very young rats (Edwards, 1977, 1981; Mayerson et al., 1985; Chang et al., 1991; Sakagami et al., 1995). Four publications reported the isolation of RPE cells from adult rats (Sheedlo et al., 1993, Wang et al., 1993; Kreppel et al., 2002; Langenfeld et al., 2015). The onset of phagocytosis of outer segments by the RPE cells occurs between P12–15 *in vivo* (Tamai and Chader, 1979). This increases the difficulty in separating the RPE cells from the photoreceptors in adult or aging animals.

In addition to protocols designed for rat tissue, we applied methods originally describing the isolation of RPE cells from human tissue. All methods describe the isolation of the RPE cells from either choroid/RPE sheets or from the complete posterior portion of the eye after removal of the retina. As mentioned above, it was not possible to peel off the RPE cells from the choroid directly (Hu and Bok, 2010) or to peel the choroid of the underlying sclera without destroying the tissue. Therefore, we incubated flattened posterior eye cups in protease solution (Figure 2B). The incubation of the eye cups in dispase (Castillo et al., 1995; Maminishkis et al., 2006; Blenkinsop et al., 2013) or papain (Kasahara et al., 2005; Bian et al., 2007) did not lead to a digestion of Bruch’s membrane, and we were not able to isolate the RPE cells (Figure 2D). Incubating the eye cups in collagenase (Gullapalli et al., 2008), collagenase + hyaluronidase (Wang et al., 1993) or trypsin (Baumgartner et al., 1989; Hunt et al., 1989; Klettner and Roider, 2008) led to a deterioration of tissue boundaries so that an isolation of RPE cells became impossible (Figure 2C). When the protease solution was used at a lower concentration, Bruch’s membrane was not properly digested and the RPE cells were still bound to the underlying Bruch’s membrane (Figure 2D).

The digestion of the whole globe with dispase (Chang et al., 1991) led to the isolation of 20,000–25,000 RPE cells per eye (Figure 2E). To achieve a higher yield of RPE cells, we modified a protocol for the use of adult tissue that was previously used to isolate retinal explants for electrophysiological measurements (Pinzon-Duarte et al., 2000; Agulhon et al., 2007). The incubation time (50 min) and the concentration (20 U/ml) of the protease were very important for the isolation of the RPE cells (Figures 2, 3). With this method we achieved a yield of 30,000–40,000 cells/eye (Figure 2F). In comparison with other protease incubation-based methods (Langenfeld et al., 2015) our culture was free of fibroblast contaminations (Figure 6). Moreover, our approach does not require an overnight incubation of the whole eye in medium to separate the retina from the underlying RPE cells (Langenfeld et al., 2015), and is therefore less time-consuming.

We were able to confirm the expression of the RPE-specific markers basigin (Philp et al., 2003), CRALBP (Huang et al., 2009), cytokeratin 18 (Johansson et al., 2010), MERTK (Feng et al., 2002; Nandrot et al., 2012), OTX-2 (Martinez-Morales et al., 2003; Housset et al., 2013), RPE65 (Huang et al., 2009; Johansson et al., 2010) and ZO-1 (Konari et al., 1995; Campbell and Humphries, 2012) by our cultured RPE cells (Figure 5). When few rat RPE cells were plated initially, the cultured cells lost their pigmentation as well as CRALBP and RPE65 expression, which are proteins involved in the visual cycle (Figures 5, 6). This has been reported for fetal and adult human RPE cells as well as immortalized RPE cell lines (Nabi et al., 1993; Wen et al., 1994; Davis et al., 1995; Vinores et al., 1995; Alge et al., 2003; Tamiya et al., 2010). RPE cells undergo a transition from an epithelial to a mesenchymal phenotype when they are cultured and lose cell-cell-contacts (Sheridan et al., 2004; Kim et al., 2008; Tamiya et al., 2010). However, de-differentiated RPE cells can re-differentiate after transplantation (Vugler et al., 2008; Lu et al., 2009; Carr et al., 2013). Furthermore, plating RPE cells at higher density maintained the RPE cells in a pigmented state for longer (Figures 6D–F).

We confirmed the secretion of collagen IV, fibronectin and laminin by the cultured RPE cells (Figure 7), which likely served as substrates in the control adhesion assays (Figure 8). This reinforced earlier studies which showed the synthesis of ECM components of Bruch’s membrane by RPE cells *in vivo* and *in vitro* (Aisenbrey et al., 2006; Afshari et al., 2010a; Sato et al., 2013).

The adhesion assays on glass coverslips coated with different ECM molecules that are found in intact Bruch’s membrane showed differential adhesion of the RPE cells in the order fibronectin > collagen IV > laminin > collagen I > glass (Figure 8). While the basement membrane of the RPE is composed of mainly collagen IV and laminin, the inner collagenous layer comprises collagen I and fibronectin, where collagen I is present in higher concentration than fibronectin (Das et al., 1990; Booij et al., 2010). Our results suggest that RPE cells adhere to a lesser extent to the collagenous layer which is exposed after choroidal new vessel removal surgery (Berger and Kaplan, 1992; Grossniklaus et al., 1994; Castellarin et al., 1998) than to the basement membrane of the RPE. This is in line with earlier studies (Del Priore and Tezel, 1998; Tezel and Del Priore, 1999; Tezel et al., 1999, 2004; Del Priore et al., 2006). Moreover, the levels of the adhesive ECM molecules collagen IV, fibronectin and laminin decline with age (Pauleikhoff et al., 1990, 1999). On the contrary, the abundance of collagen I increases (Ramrattan et al., 1994), making aged Bruch’s membrane less adhesive. These experiments also demonstrate that the adult RPE cells isolated using our method would provide a good aged-matched tool for the study of diseases related to RPE cells.

In summary, we present here a novel and reliable culture method for rat RPE cells from adult animals using papain. In comparison to other already published protocols it yields more cells and is less time-consuming. The cultured cells can be used to study the behavior of RPE cells *in vitro* and *in vivo* as it avoids rejection issues. The protocol can easily be adapted for other species such as mice. This method will benefit researchers studying therapeutic targets, test the effects of drugs in preclinical setups or perform *in vitro* and *in vivo* transplantation experiments of adult cells to study retinal diseases.

## Conflict of Interest Statement

The authors declare that the research was conducted in the absence of any commercial or financial relationships that could be construed as a potential conflict of interest.

## Abbreviations

AMD: age-related macular degeneration
CRALBP: cellular retinaldehyde binding protein
DIV: days *in vitro*
ECM: extracellular matrix
FBS: fetal bovine serum
MERTK: c-mer proto-oncogene tyrosine kinase
OTX-2: orthodenticle homeobox 2
PBS: phosphate-buffered saline
PDL: poly-D-lysine
PFA: paraformaldehyde
RT: room temperature
RCS: Royal College of Surgeon’s
RPE: retinal pigment epithelium
RPE65: 65 kDa retinal pigment epithelium specific protein
SD: Sprague Dawley
SEM: standard error of the mean
ZO-1: zona occludens protein 1.
